# COVID-19 and psychiatric inpatient admissions at a Saudi tertiary hospital, 2017-2024: an interrupted time-series study

**DOI:** 10.3389/fpsyt.2026.1922988

**Published:** 2026-09-30

**Authors:** Mohammed T. Alotaibi, Fahad M. Alotaibi, Malek I. Almatroudi, Enas M. Aljohani, Fatimah A. Alqahtani, Abdulkarim M. Almudhi, Dyna K. Alhajri, Nahaa E. Alsubaie, Afnan A. AlMarshedi, Youssef F. Alshehri

**Affiliations:** 1 Department of Psychiatry, Prince Sultan Military Medical City, Riyadh, Saudi Arabia; 2 Department of Mathematics and Statistics, University College of Khurma, Taif University, Taif, Saudi Arabia

**Keywords:** COVID-19, interrupted time series, length of stay, mental health services, psychiatric inpatient admissions, Saudi Arabia, segmented regression, substance use disorders

## Abstract

**Introduction:**

Psychiatric inpatient admissions decreased in many countries during the COVID-19 pandemic. Most studies used a year or less of pre-pandemic data and compared the two periods directly, which cannot separate a pandemic effect from the increase that predated it, and evidence from the Middle East is limited. This study examined changes in psychiatric admission volume across the COVID-19 pandemic, with diagnostic case-mix and length of stay (LOS) examined as secondary outcomes.

**Methods:**

This interrupted time-series analysis used eight years of inpatient records from Prince Sultan Military Medical City, a tertiary hospital in Riyadh, Saudi Arabia. All 1551 adult psychiatric admissions between January 2017 and November 2024 were assigned to pre-COVID (January 2017 to February 2020), during-COVID (March 2020 to December 2021), and post-COVID (January 2022 to November 2024) periods. Monthly admission counts were modeled by segmented Poisson regression. Diagnostic case-mix was assessed by chi-square tests and length of stay by a linear mixed-effects model.

**Results:**

Before the pandemic, admissions were increasing by 1.7% per month (IRR 1.017; 95% CI 1.009-1.024), a trend concentrated in the first recording year (2017). With the baseline restricted to 2018 onward, this trend was flat. At onset admissions decreased by 31.5% (IRR 0.685; 95% CI 0.564-0.831), a reduction that persisted across baseline specifications. Monthly volume then remained near the pre-pandemic mean of 16 to 17 through 2024. Diagnostic composition differed across periods. Admissions for substance use disorders increased from 9.0% to 14.3% in the post-COVID period (absolute difference 5.3 percentage points; 95% CI 1.6-9.0), and admissions for mood disorders decreased from 37.4% to 30.5%. Length of stay showed no significant change.

**Conclusion:**

At this single center, admissions fell at onset and then remained near their pre-pandemic level through 2024. The diagnostic and sociodemographic differences were small and should be regarded as exploratory. Multicenter studies are needed to confirm these findings and to inform mental health service planning for future public health emergencies.

## 1 Introduction

The COVID-19 pandemic disrupted the delivery and use of mental health care worldwide. Movement restrictions and the redeployment of hospital staff and beds altered how and when patients reached care. Inpatient psychiatric admissions, which depend on both the severity of illness and access to hospital beds, decreased in many countries during the first months of the pandemic. A meta-analysis of 128 studies estimated a 25% reduction in psychiatric hospital use (risk ratio 0.75; 95% CI 0.67-0.85) (1), with national figures ranging from an 11% decrease in Denmark (2) to 41% in Italy (3). Part of this variation has been attributed to differences in lockdown stringency and in the reorganization of health services (4, 5).

The change was not uniform across settings or diagnostic groups. In Spain, total emergency presentations decreased while the proportion of patients requiring admission increased (6). In Berlin, overall admissions were unchanged, although presentations involving suicide attempts increased (7). A review of self-harm presentations found an overall decrease, with increases limited to adolescents (8). Interpretation of these reports is constrained by their design. Most relied on a year or less of pre-pandemic data and on direct before-and-after comparisons, which cannot separate a pandemic effect from a trend already present (1, 3, 5, 9). Most also ended during the first or second wave, leaving the durability of the change unknown (4).

Evidence from the Middle East is limited and shares these constraints. Saudi Arabia introduced among the most stringent restrictions internationally in 2020, including 24-hour curfews in major cities and the suspension of domestic travel and non-essential services. The available reports point in different directions. Psychiatric emergency visits increased by 25.66% across 2 Ministry of National Guard Health Affairs centers in Riyadh and Jeddah (10), whereas substance use admissions at an addiction hospital in Dammam decreased by approximately 70% during the lockdown (11). Neither study used a design that separates a pandemic effect from an underlying trend, and neither extended beyond 2021.

The present study aimed to characterize the trajectory of psychiatric inpatient admissions at Prince Sultan Military Medical City, a tertiary hospital in Riyadh, across the COVID-19 pandemic. It sought to determine whether the onset of the pandemic coincided with a change in admission rates and whether the pre-pandemic increase resumed afterward. A further aim was to establish whether the diagnostic composition and length of stay (LOS) of admissions changed.

## 2 Materials and methods

### 2.1 Study design and setting

This retrospective cohort study examined psychiatric inpatient admissions at Prince Sultan Military Medical City (PSMMC), a tertiary hospital in Riyadh, Saudi Arabia. PSMMC provides care to a defined population of eligible beneficiaries and their dependents. Patients seen in Ministry of Health or private-sector facilities are not represented. The department has 35 inpatient beds (20 for men and 15 for women) and outpatient clinics. It moved to a purpose-built facility in 2017, and the bed complement and clinical workflow were unchanged across the study period, with a modest increase in staffing. Most admissions originated from the emergency room (81.3%) and outpatient clinics (12.9%), with the remainder from internal transfers and direct admissions. The study was approved by the PSMMC Research Ethics Committee (approval number E-2828). Individual consent was waived owing to the retrospective design and use of deidentified data. Reporting follows the Strengthening the Reporting of Observational Studies in Epidemiology (STROBE) guideline for cohort studies (12).

### 2.2 Participants

All psychiatric inpatient admissions of adults aged 18 years or older between January 1, 2017, and November 30, 2024, were included. The unit of analysis was the admission, and each admission by a patient with multiple hospitalizations was counted separately. Admissions for nonpsychiatric reasons, those with incomplete records, stays shorter than 24 hours, and court-ordered or administrative admissions were excluded. Court-ordered admissions follow judicial rather than clinical pathways and do not reflect voluntary service demand, and administrative admissions lack a primary psychiatric indication. Both would introduce nonclinical variability into the monthly counts. These categories were identified at data extraction and formed a small minority of admissions at this general, non-forensic unit.

### 2.3 COVID-19 period definitions

Admissions were classified into 3 prespecified periods: pre-COVID (January 2017 to February 2020), during-COVID (March 2020 to December 2021), and post-COVID (January 2022 to November 2024). March 2020 was chosen because the Saudi Ministry of Health confirmed the first case on March 2, 2020, and public health restrictions were implemented within that month. The boundary between the during-COVID and post-COVID periods (January 2022) marks the lifting of the principal movement restrictions and the transition to the Omicron phase, after which curfews and large-scale closures were no longer in force. This three-period classification was used only for descriptive case-mix and length-of-stay comparisons. The segmented regression modeled a single interruption at March 2020, coded as the first pandemic-affected month.

### 2.4 Outcomes and variables

The primary outcome was monthly admission count. Secondary outcomes were diagnostic case-mix and LOS. Primary diagnosis was classified using the International Statistical Classification of Diseases and Related Health Problems, Tenth Revision (ICD-10) (13) into 5 diagnostic groups: schizophrenia-spectrum disorders (F20-F29), mood disorders (F30-F39), substance use disorders (F10-F19), anxiety and trauma-related disorders (F40-F48), and other psychiatric disorders. LOS was calculated in days and categorized as short (≤30 days) or long (>30 days). Sociodemographic variables included age, sex, marital status, education, and employment status.

### 2.5 Statistical analysis

Analyses were performed in IBM SPSS Statistics version 30 (IBM Corp). Sociodemographic characteristics were compared across the 3 periods using χ² tests with Cramér V as the effect-size measure for categorical variables and analysis of variance for age. Where expected cell counts were small, such as unknown employment status during the pandemic, the comparisons were interpreted with caution. The primary outcome, monthly admission count, was analyzed at the aggregate level by segmented Poisson regression (interrupted time-series analysis), following the approach of Wagner et al. (14). The model included a continuous term for time (the underlying trend), a binary term for the pandemic period (the level change at March 2020), and a term for time since onset (the change in trend), and was fitted by generalized linear modeling with a Poisson distribution and log link. Incidence rate ratios (IRRs) with 95% confidence intervals were obtained by exponentiating the coefficients. Residual autocorrelation was examined with the Durbin-Watson statistic, the autocorrelation and partial autocorrelation functions, and the Ljung-Box test. Low-order autocorrelation was present (lag-1 autocorrelation 0.33; Ljung-Box P = .002). Inference was based on Newey-West standard errors, which allow for autocorrelation, with a maximum lag of 3. A generalized estimating equation with a first-order autoregressive working correlation (estimated autocorrelation 0.31) gave the same result. The level change remained significant under both (Newey-West IRR 0.685; 95% CI 0.51-0.91; P = .01; GEE IRR 0.687; 95% CI 0.54-0.88; **Supplementary Table 1**). Overdispersion was modest (Pearson dispersion 1.52) and does not bias the Poisson point estimates. The quasi-Poisson and negative binomial specifications (alpha = 0.03), together with the robust standard errors above, preserved the direction and significance of all terms, and the Poisson model was retained for its interpretable estimates (**Supplementary Table 1**). Seasonality was tested by adding annual and semiannual harmonic terms, which did not improve fit (likelihood-ratio χ²(4) = 4.90; P = .30), and no seasonal term was retained. The dependence of the level change on the assumed pre-pandemic trajectory was examined by refitting the model with the baseline restricted to 2018 onward and to 2019 onward and with a quadratic baseline term (**Supplementary Table 1**). The interruption point was set at March 2020. Placing it at April 2020 gave a similar level change (IRR 0.70; 95% CI 0.58-0.85; **Supplementary Table 1**). The secondary outcomes, diagnostic case-mix and length of stay, were analyzed at the level of the individual admission. The distribution of the 5 diagnostic groups was compared across periods by χ² test. Length of stay was right-skewed and was log10-transformed, then analyzed by a linear mixed-effects model with period as a fixed effect and a random intercept for the patient, estimated by restricted maximum likelihood with Satterthwaite degrees of freedom. The random intercept was included because 294 of the 971 patients (30.3%) contributed more than one admission, up to 17. Estimated marginal means are reported after back-transformation to days. The proportion of long stays (>30 days) was compared across periods by χ² test. All tests were 2-sided, with α = .05.

## 3 Results

### 3.1 Sociodemographic characteristics

Of the 1551 admissions, 645 (41.6%) occurred in the pre-COVID period, 361 (23.3%) during-COVID, and 545 (35.1%) post-COVID. Sociodemographic characteristics by period are shown in **Table 1**. Most admissions were of men (1088; 70.2%), and the male proportion was highest during the pandemic (66.7% pre-COVID, 76.2% during, 70.3% post-COVID; P = .007). The proportion of single patients increased from 48.5% pre-COVID to 56.3% post-COVID (P = .04). Educational attainment differed across periods (P <.001): the low-education proportion decreased from 15.0% to 9.0% and the secondary-education proportion increased from 47.9% to 58.2%. Unemployment was higher post-COVID than pre-COVID (45.9% vs 41.9%; P <.001). Age did not differ across periods (mean [SD], 35.40 [11.75] pre-COVID, 34.33 [10.75] during, 35.53 [12.48] post-COVID; P = .27). Effect sizes were small for all variables (Cramér V ≤ 0.17).

**Table 1:** Sociodemographic characteristics of psychiatric inpatient admissions by COVID-19 period.

		Overall	Pre-COVID	During-COVID	Post-COVID		
Characteristic		(N = 1551)	(n = 645)	(n = 361)	(n = 545)	P value	Cramér V
Sex, No. (%)						.007	.080
	Male	1088 (70.2)	430 (66.7)	275 (76.2)	383 (70.3)		
	Female	463 (29.8)	215 (33.3)	86 (23.8)	162 (29.7)		
Marital status, No. (%)						.042	.057
	Single	817 (52.7)	313 (48.5)	197 (54.6)	307 (56.3)		
	Married	469 (30.2)	221 (34.3)	103 (28.5)	145 (26.6)		
	Previously married	265 (17.1)	111 (17.2)	61 (16.9)	93 (17.1)		
Education level, No. (%)						<.001	.130
	Higher education	398 (25.7)	160 (24.8)	104 (28.8)	134 (24.6)		
	Secondary education	817 (52.7)	309 (47.9)	191 (52.9)	317 (58.2)		
	Low education	175 (11.3)	97 (15.0)	29 (8.0)	49 (9.0)		
	Unknown	161 (10.4)	79 (12.2)	37 (10.2)	45 (8.3)		
Employment status, No. (%)						<.001	.166
	Employed	408 (26.3)	177 (27.4)	101 (28.0)	130 (23.9)		
	Unemployed	664 (42.8)	270 (41.9)	144 (39.9)	250 (45.9)		
	Retired	265 (17.1)	92 (14.3)	70 (19.4)	103 (18.9)		
	Student	146 (9.4)	58 (9.0)	38 (10.5)	50 (9.2)		
	Unknown	66 (4.3)	48 (7.4)	6 (1.7)	12 (2.2)		
Age, mean (SD), y		35.10 (11.80)	35.40 (11.75)	34.33 (10.75)	35.53 (12.48)	.274	

*V*, Cramér *V*.

Data are presented as No. (%) unless otherwise indicated.

*P* values are from Pearson χ² tests for categorical variables and analysis of variance for age.

### 3.2 Admission volume

Annual and monthly admission counts are provided in **Supplementary Tables 2**, **3**. During the pre-COVID period, admissions were concentrated in 2018 (35.7%) and 2019 (34.6%). During the COVID period, 59.8% occurred in 2021, partly because 2020 contributed only 10 months to this window (March to December) and restrictions were most stringent that year. Post-COVID admissions were distributed evenly across 2022 (31.7%), 2023 (35.4%), and 2024 (32.8%). Segmented Poisson regression identified three components of the monthly series (**Table 2**; **Figure 1**). The pre-pandemic trend was a monthly increase of 1.7% (IRR 1.017; 95% CI 1.009-1.024; P <.001). Pandemic onset was associated with a level reduction of 31.5% (IRR 0.685; 95% CI 0.564-0.831; P <.001). The slope-change term was significant (IRR 0.984; 95% CI 0.976-0.992; P <.001), although the net post-onset slope did not differ from zero (IRR 1.001; 95% CI 0.997-1.005; P = .69). After the initial reduction, admissions formed a plateau rather than resuming their pre-pandemic increase. In absolute terms, monthly admissions were similar across the three periods (means 17.0 pre-COVID, 16.4 during-COVID, and 15.6 post-COVID). The 31.5% level reduction therefore reflects a departure from the rising pre-pandemic trend rather than a decline in absolute volume. That trend was concentrated in the first baseline year. Recorded volume in 2017 (146 admissions) was lower than in 2018 (230) and 2019 (223) because 2017 was the first year of the institution’s electronic medical record and admission capture was still incomplete. With 2017 excluded, the monthly increase was 0.3% rather than 1.7%. The level reduction persisted when the baseline was restricted to 2018 onward (IRR 0.78; 95% CI 0.63-0.97) or to 2019 onward (IRR 0.72; 95% CI 0.55-0.93) and under a quadratic baseline (IRR 0.62; 95% CI 0.50-0.77), although its estimated magnitude depended on the assumed pre-pandemic trajectory (**Supplementary Table 1**).

**Table 2:** Segmented poisson regression analysis of monthly psychiatric inpatient admissions.

Predictor	B	IRR	95% CI	Wald χ²	P Value
Pre-pandemic monthly trend (time)	0.017	1.017	1.009-1.024	20.84	<.001
COVID period (level change)	−0.379	0.685	0.564-0.831	14.77	<.001
Post-COVID time (slope change)	−0.016	0.984	0.976-0.992	14.39	<.001

IRR, incidence rate ratio.

The level-change term contrasts the post-onset period with the pre-pandemic period.

The slope-change term is the change relative to the pre-pandemic trend. The net post-COVID monthly slope was IRR 1.001 (95% CI, 0.997-1.005).

**Figure 1:**
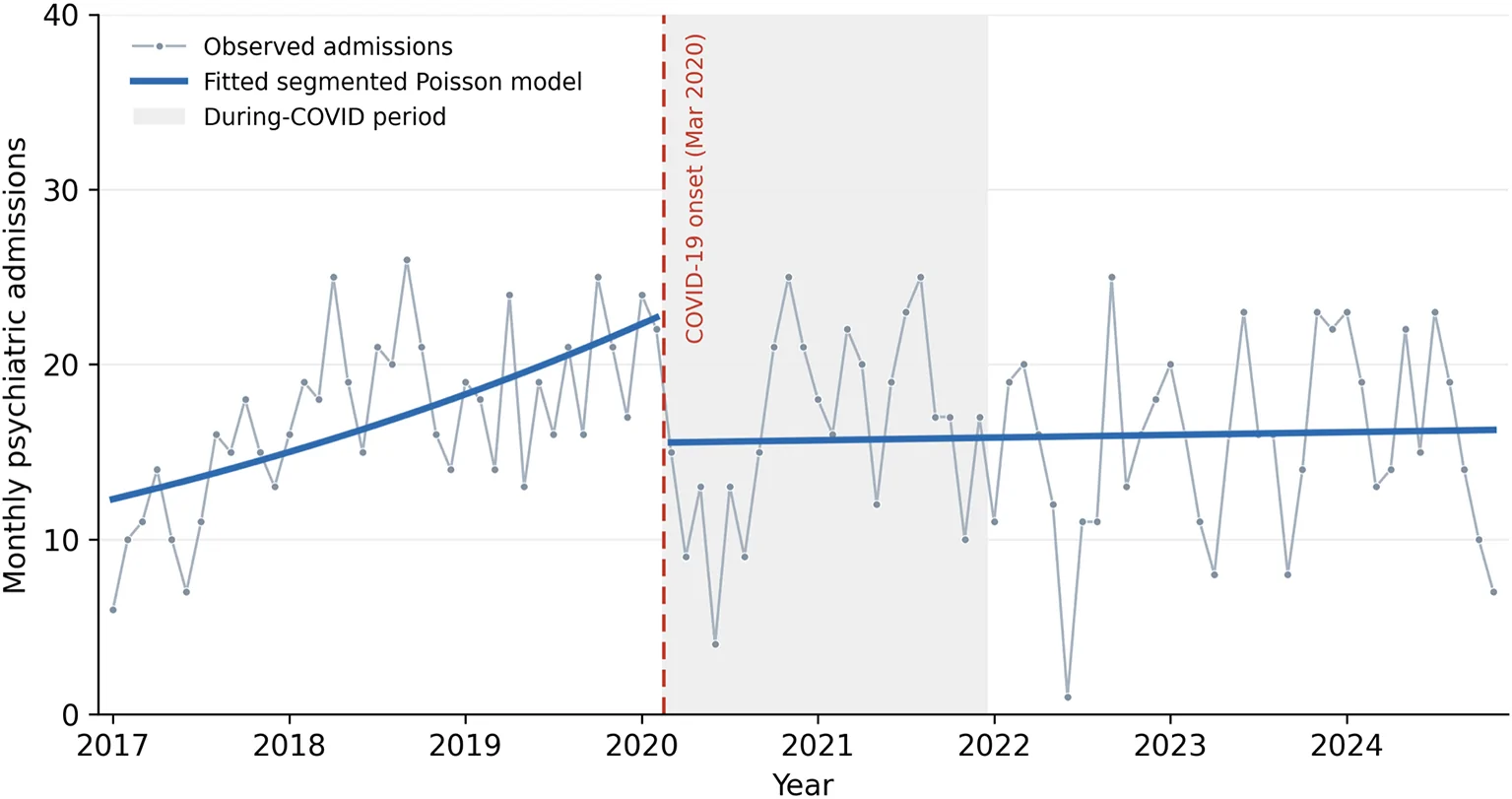
Observed monthly psychiatric inpatient admissions and fitted segmented poisson regression model across COVID-19 periods.

### 3.3 Diagnostic distribution

The distribution of diagnostic groups differed across periods, although the overall effect was small (χ²(8) = 17.93; P = .02; Cramér V = 0.08; **Table 3**; **Supplementary Figure 1**). Schizophrenia-spectrum disorders were the most common category in every period (39.4% pre-COVID, 43.8% during, 38.2% post-COVID). Mood disorders proportion decreased from 37.4% pre-COVID to 31.0% during the pandemic and was 30.5% post-COVID. The substance use disorder proportion was 9.0% pre-COVID and 9.1% during, then increased to 14.3% post-COVID (difference vs pre-COVID, 5.3 percentage points; 95% CI, 1.6-9.0).

**Table 3:** Distribution of psychiatric diagnostic groups by COVID-19 period.

Diagnostic group	Pre-COVID, no. (%)	During-COVID, no. (%)	Post-COVID, no. (%)
Schizophrenia-spectrum disorders	254 (39.4)	158 (43.8)	208 (38.2)
Mood disorders	241 (37.4)	112 (31.0)	166 (30.5)
Substance use disorders	58 (9.0)	33 (9.1)	78 (14.3)
Anxiety/trauma-related disorders	39 (6.0)	21 (5.8)	37 (6.8)
Other psychiatric disorders	53 (8.2)	37 (10.2)	56 (10.3)

χ²(8) = 17.93; P = .02; Cramér V = 0.08.

### 3.4 Length of stay

Length of stay was right-skewed in every period (mean [SD], 26.30 [26.36] days pre-COVID, 24.87 [35.54] during, 29.92 [43.61] post-COVID; medians 21, 18, and 20 days; **Supplementary Table 4**). On log10-transformed values, the linear mixed-effects model showed no period effect (F(2, 1503.85) = 1.79; P = .17). Back-transformed geometric mean length of stay was 18.6 days pre-COVID (95% CI 17.3-19.9), 17.0 during (15.6-18.5), and 18.6 post-COVID (17.3-20.0). Between-patient variance was significant (random intercept 0.05; P <.001). The proportion of long stays (>30 days) did differ across periods (27.0% pre-COVID, 19.9% during, 28.8% post-COVID; χ²(2) = 9.44; P = .009; **Supplementary Table 5**; **Supplementary Figure 2**).

## 4 Discussion

Psychiatric admissions to this tertiary hospital decreased by 31.5% at the onset of the pandemic and then remained near their pre-pandemic level through 2024. Before COVID-19, recorded admissions had been increasing by about 1.7% each month. This increase was concentrated in 2017, the first year of the electronic record. With the baseline restricted to 2018 onward, the trend was essentially flat (IRR 1.003). The level reduction at onset held under this restriction. The conclusion that pre-pandemic growth was interrupted depends heavily on the first recording year and should be read with caution. The extended record still allows the change at onset to be separated from the pre-existing trajectory, which short before-and-after comparisons cannot do. Diagnostic case-mix differed across periods, though the effect size was small. Substance use disorder admissions increased from 9.0% to 14.3% after restrictions were lifted, and mood disorder admissions decreased from 37.4% to 30.5% and remained below their pre-pandemic level. Length of stay showed no significant change.

At our site the reduction was in total admissions, and this was not universal. In a Berlin emergency department, overall presentations and admissions did not change significantly, even though inpatient bed capacity decreased by a third (7). In a Spanish service, emergency presentations decreased by 37.9%, yet a higher proportion of those who presented were admitted, and the reduction in inpatient admissions was therefore smaller than in presentations (6). Our finding instead resembles the clear reductions in total psychiatric admissions reported in Italy and Brazil (3, 15), under some of the most stringent and prolonged restrictions imposed during the pandemic (16).

The admissions did not recover, and our follow-up to late 2024 sets this apart from the main comparators, which ended in 2021. The Italian CoMeH study, covering about 6 million people and using the same interrupted time-series approach, found that first psychiatric admissions resumed a moderate upward trend after the initial drop yet had not reached pre-pandemic levels by December 2021 (17). A review of 177 European studies found that service use increased through 2021 without returning to pre-pandemic levels for some services (4). Our study shows no such recovery. Annual admissions remained near 180, below the 2018 and 2019 counts of 230 and 223, with no upward movement across three post-pandemic years. The data do not show where the missing admissions went. During the pandemic the route of admission shifted. A larger share arrived through the emergency room, from 78.9% to 85.6%, and fewer through outpatient clinics, from 15.5% to 8.3%. The fall in outpatient referrals is what reduced access to those clinics would produce. One possibility is remote care. The Saudi Ministry of Health expanded telemental health during the first year of the pandemic (18), and Glock et al. reported a more than sevenfold increase in telemedicine use during the pandemic (1). Whether this substitution accounts for the sustained reduction cannot be tested without site-level telehealth records.

The demographic profile changed only modestly. The hospital serves a predominantly male beneficiary population, so the sex distribution and its changes reflect that population rather than a general trend. Male admissions rose during the acute phase, from 66.7% to 76.2%, alongside the rise in schizophrenia-spectrum admissions, a male-predominant group. Both changes are small, and we interpret them cautiously given the population served. Comparisons with other settings are limited by differences in beneficiary populations and case-mix. The closest regional comparison, Qamruddin et al. in the United Arab Emirates, found no difference in sex, age, or marital status (20). Davies and Hogarth, in a UK service, reported a stable gender mix, with only male schizophrenia admissions increasing early in lockdown (19). Rodrigues et al, in a Lisbon ward where total admissions fell by 30.7%, close to our figure, found no difference in gender or age (21). The unemployed proportion increased from 41.9% to 45.9%, during a period when economic disruption concentrated on insecure workers (22) and unemployment carries a higher burden of mental illness (23). Admissions from patients with low education decreased. Petrelli et al. reported the same direction in Italy, where the reduction in mental health service use was steepest in the most deprived areas (24). In Saudi Arabia, stigma and low mental health awareness are the most commonly reported barriers to care (25), and reduced access during the pandemic would have reached the most disadvantaged patients first. The proportion of single patients increased from 48.5% in the pre-COVID period to 56.3% in the post-COVID period, while that of married patients decreased from 34.3% to 26.6%. Married patients report stronger social support than unmarried patients (26), and both limited social support and single status are associated with a higher risk of involuntary psychiatric admission, which Walker et al. attribute partly to the role of family in facilitating voluntary help-seeking (27). In Saudi Arabia, limited family support is itself among the reported obstacles to mental health help-seeking (25), likely amplified as community services contracted during the pandemic. The association is descriptive and does not imply a difference in admission risk by marital status. The profile was otherwise stable, which indicates that the decrease in admissions was spread across groups rather than concentrated in one.

The schizophrenia-spectrum proportion increased during the pandemic, from 39.4% to 43.8%, then returned to 38.2%. Psychotic disorders more often reach inpatient care through emergency and involuntary routes, which were less sensitive to the help-seeking avoidance of lockdown. Fasshauer et al, analyzing claims data from a German-wide hospital network, found that the proportion of urgent admissions increased from 60.6% in 2019 to 62.9% in 2020, with a parallel increase in involuntary admissions, even though the absolute number of urgent admissions decreased (28). Rodrigues et al, in a Lisbon acute ward where total admissions decreased by 30.7%, found that schizophrenia had the smallest reduction of any diagnostic group (21). Kelbrick et al, in a UK inpatient unit, recorded higher admission rates for both first-episode and repeated-episode psychosis in the 12 months after onset (29). The mood disorder proportion decreased over the same period, from 37.4% to 31.0% and then 30.5%. Affective presentations may have been more amenable to outpatient and remote management when inpatient access was constrained, and Dionisie et al. reported that affective admissions decreased during lockdown at a large Romanian teaching hospital (30).

Substance use disorder admissions were the clearest diagnostic change, at 9.0% pre-COVID, 9.1% during, and 14.3% post-COVID. The increase was concentrated after restrictions ended, and admissions did not fall during the acute phase. Evidence from general psychiatric services is mixed. In a general psychiatric service in Jeddah, Jahlan et al. reported that substance use referrals rose during lockdown (31), whereas at a Lisbon acute ward Rodrigues et al. found that substance use admissions fell by more than half, the largest reduction of any diagnostic group (21). Our pattern matched neither. Addiction-specialist data describe a different service but help interpret the timing. At an addiction center in Dammam, Ramadan and Batwa found that substance use admissions fell by about 70% during the curfew and rose once it was lifted, concentrated among young adults with amphetamine use disorder (11). Our general-hospital series shows the same timing, a fall during restrictions and a rise afterward. Pandemic-related stress and uncertainty may have raised substance use, while restricted drug access during the curfew, followed by its restoration, plausibly accounts for the post-COVID timing. Whether the increase reflects deferred presentations returning, a genuine rise in substance use, or both cannot be separated here, and data from other Saudi facilities would be needed to establish a system-level trend.

Average length of stay did not change across periods, whereas the proportion of stays longer than 30 days fell during the pandemic and recovered afterward. The two findings are not contradictory. The average describes the typical admission, which did not change, whereas the long-stay proportion describes only the longest-staying patients, where the temporary decrease occurred. A temporary decrease in long stays is what faster discharge would produce when bed pressure and infection control take priority, and other systems also recorded shorter stays. Fasshauer et al. found that mean stay in a German hospital network decreased from 14.7 to 9.8 days during the first outbreak (9). Russolillo et al, by contrast, found no change in Vancouver (32), whereas Boldrini et al. found the opposite in Italy, where long stays increased by 63% during lockdown because fewer patients were admitted while those who were proved harder to discharge (3). After the pandemic, our long-stay proportion returned to within 2 percentage points of its starting value, a difference that was not significant.

### 4.1 Strengths and limitations

Comparisons across the pandemic-admissions literature are complicated by heterogeneity in the services studied. Settings vary in the populations they serve and in how care is organized and reached. They also vary in whether admissions are acute or planned and voluntary or involuntary. Our unit admits mainly through the emergency room (81.3%), so its counts reflect acute, largely unplanned demand rather than elective activity. This may explain why our level reduction resembles the clear drops reported in acute services and differs from settings dominated by planned admissions. Where our findings diverge from others, differences in case definition and in the acute or elective mix are a more likely explanation than a true difference in the pandemic’s effect. The main contribution of this study is longitudinal evidence, from an extended pre-pandemic baseline, that admissions at one Saudi tertiary service fell to a lower level and did not recover through 2024. This bears on how psychiatric capacity and access are planned for future emergencies.

The principal strength of this study is its methodology, which is an extended pre-pandemic observation period. Studies of pandemic-era admissions have typically used a year or less of earlier data and compared the two periods directly, which cannot distinguish a pandemic effect from the increase that predated it. The 8 years of monthly data analyzed here allowed that distinction, and the level reduction at onset persisted across restricted and nonlinear baseline specifications, although its estimated magnitude depended on the assumed pre-pandemic trajectory. That said, it is not without limitations. Several should be considered. First, the data come from a single tertiary hospital serving a defined beneficiary population that is predominantly male, reflected in the 70.2% male admissions, which shapes the diagnostic case-mix and limits generalizability to the wider Saudi population. Beneficiaries may also obtain psychiatric care in Ministry of Health or private-sector facilities, so these admissions capture only part of their care. We report admission counts rather than population rates, because a stable denominator for the eligible population across the study period was not available. If that population changed in size, counts would not fully track admission rates. Second, the bed complement (35 beds) and clinical workflow were unchanged across the study period, which makes a supply-side explanation for the level reduction, such as ward conversion or bed repurposing, unlikely. Ward-level occupancy statistics were not retained and could not be examined directly. Third, the interrupted time-series design is observational, and although admissions changed at the pandemic onset, this cannot be attributed to the pandemic with certainty, as it coincided with widespread changes in health-care delivery. Fourth, the analysis relied on routinely collected administrative data. Diagnoses were not independently validated, and illness severity and involuntary status were unavailable. The diagnostic and demographic comparisons were not corrected for multiple testing and carried small effect sizes (case-mix Cramér V = 0.08), and those shifts should be regarded as exploratory. The unit admits only about 16 to 17 patients per month, and monthly counts vary considerably, so relative changes in specific subgroups correspond to small absolute numbers. Diagnosis-specific time-series models would be underpowered, and the case-mix comparisons are descriptive rather than evidence of causal effects on particular diagnoses. Finally, admission capture in 2017 was incomplete because the electronic medical record was introduced that year. Restricting the baseline to 2018 onward did not change the level reduction (**Supplementary Table 1**).

## 5 Conclusion and future directions

The onset of the COVID-19 pandemic was associated with a reduction in psychiatric admissions relative to their rising pre-pandemic trend. In absolute terms, monthly admissions remained near their pre-pandemic average through 2024, and the earlier increase did not resume. Admissions for substance use disorders increased and those for mood disorders decreased over the same period, by small margins, and these shifts should be regarded as exploratory. The size of the onset reduction depended on the pre-pandemic baseline used, and the apparent interruption of earlier growth rested largely on the first recording year. Findings from a single center should contribute to, rather than by themselves determine, mental health service planning. Future multisite studies incorporating telehealth and outpatient utilization data are needed to capture possible shifts toward remote or community-based care and to support broader generalizability of these findings. Such data would also strengthen preparation for future public health emergencies.

## Data Availability

The raw data supporting the conclusions of this article will be made available by the authors, without undue reservation.
