# Supplementary material for: COVID-19 and psychiatric inpatient admissions at a Saudi tertiary hospital, 2017-2024: an interrupted time-series study
Source: Front Psychiatry. 2026 Sep 30;17:1922988. doi: 10.3389/fpsyt.2026.1922988 (PMC13662059; doi:10.3389/fpsyt.2026.1922988)
Supplement: Supplementary file 1 [file Table1.docx]

**Supplementary Material**

**Contents**

Supplementary Table 1. Sensitivity analyses for the segmented Poisson model.

Supplementary Table 2. Distribution of psychiatric inpatient admissions by year across COVID-19 periods.

Supplementary Table 3. Distribution of psychiatric inpatient admissions by month across COVID-19 periods.

Supplementary Table 4. Descriptive statistics for length of stay across COVID-19 periods.

Supplementary Table 5. Distribution of long hospital stays (>30 days) by COVID-19 period.

Supplementary Figure 1. Distribution of psychiatric diagnostic groups across COVID-19 periods.

Supplementary Figure 2. Length-of-stay categories across COVID-19 periods.

*This Supplementary Material has been provided by the authors to give readers additional information about the work.*

**Supplementary Table 1. Sensitivity analyses for the segmented Poisson model**

**Panel A. Robustness to overdispersion and distributional assumptions**

| **Term** | **Poisson IRR (95% CI)** | **Quasi-Poisson IRR (95% CI)** | **Negative binomial IRR (95% CI)** |
| --- | --- | --- | --- |
| Pre-pandemic monthly trend | 1.017 (1.009-1.024) | 1.017 (1.008-1.026) | 1.017 (1.008-1.026) |
| Level change at COVID onset | 0.685 (0.564-0.831) | 0.685 (0.539-0.870) | 0.678 (0.531-0.867) |
| Post-COVID slope change | 0.984 (0.976-0.992) | 0.984 (0.975-0.994) | 0.984 (0.974-0.994) |

*IRR, incidence rate ratio; CI, confidence interval. Quasi-Poisson uses a Pearson scale of 1.53; the negative binomial estimated a dispersion of α = 0.03. All three terms remained significant under both specifications. Adding annual and semiannual harmonic terms did not improve model fit (likelihood-ratio χ²(4) = 4.90; P = .30), so no seasonal term was retained.*

**Panel B. Specification of the pre-pandemic baseline trend**

| **Pre-pandemic baseline** | **Pre-pandemic trend (IRR)** | **Level change IRR (95% CI)** | **P** |
| --- | --- | --- | --- |
| Full series, 2017-2020 (primary) | 1.017 | 0.685 (0.564-0.831) | <.001 |
| Restricted to 2018 onward | 1.003 | 0.781 (0.630-0.968) | .024 |
| Restricted to 2019 onward | 1.019 | 0.716 (0.554-0.925) | .011 |
| Quadratic baseline term | — | 0.617 (0.495-0.769) | <.001 |
| Interruption at April 2020 | 1.017 | 0.704 (0.580-0.854) | <.001 |
| Newey-West robust SE | — | 0.685 (0.511-0.919) | .012 |

*The level reduction at COVID-19 onset remained statistically significant under every baseline specification (restricted windows and a quadratic baseline). Its estimated magnitude depended on the assumed pre-pandemic trajectory, which was steepest in the first baseline year (2017).*

**Supplementary Table 2. Distribution of psychiatric inpatient admissions by year across COVID-19 periods**

| **COVID period** | **Year** | **No.** | **%** |
| --- | --- | --- | --- |
| Pre-COVID (Jan 2017 - Feb 2020) | 2017 | 146 | 22.6 |
|  | 2018 | 230 | 35.7 |
|  | 2019 | 223 | 34.6 |
|  | 2020 (Jan-Feb) | 46 | 7.1 |
|  | Total | 645 | 100.0 |
| During-COVID (Mar 2020 - Dec 2021) | 2020 (Mar-Dec) | 145 | 40.2 |
|  | 2021 | 216 | 59.8 |
|  | Total | 361 | 100.0 |
| Post-COVID (Jan 2022 - Nov 2024) | 2022 | 173 | 31.7 |
|  | 2023 | 193 | 35.4 |
|  | 2024 | 179 | 32.8 |
|  | Total | 545 | 100.0 |

*Percentages are within each COVID period. Overall, the pre-COVID period contributed 645 admissions (41.6%), during-COVID 361 (23.3%), and post-COVID 545 (35.1%) of the 1551 total.*

**Supplementary Table 3. Distribution of psychiatric inpatient admissions by month across COVID-19 periods**

| **COVID period** | **Month** | **No.** | **%** |
| --- | --- | --- | --- |
| Pre-COVID | 2017-01 | 6 | 0.9 |
|  | 2017-02 | 10 | 1.6 |
|  | 2017-03 | 11 | 1.7 |
|  | 2017-04 | 14 | 2.2 |
|  | 2017-05 | 10 | 1.6 |
|  | 2017-06 | 7 | 1.1 |
|  | 2017-07 | 11 | 1.7 |
|  | 2017-08 | 16 | 2.5 |
|  | 2017-09 | 15 | 2.3 |
|  | 2017-10 | 18 | 2.8 |
|  | 2017-11 | 15 | 2.3 |
|  | 2017-12 | 13 | 2.0 |
|  | 2018-01 | 16 | 2.5 |
|  | 2018-02 | 19 | 2.9 |
|  | 2018-03 | 18 | 2.8 |
|  | 2018-04 | 25 | 3.9 |
|  | 2018-05 | 19 | 2.9 |
|  | 2018-06 | 15 | 2.3 |
|  | 2018-07 | 21 | 3.3 |
|  | 2018-08 | 20 | 3.1 |
|  | 2018-09 | 26 | 4.0 |
|  | 2018-10 | 21 | 3.3 |
|  | 2018-11 | 16 | 2.5 |
|  | 2018-12 | 14 | 2.2 |
|  | 2019-01 | 19 | 2.9 |
|  | 2019-02 | 18 | 2.8 |
|  | 2019-03 | 14 | 2.2 |
|  | 2019-04 | 24 | 3.7 |
|  | 2019-05 | 13 | 2.0 |
|  | 2019-06 | 19 | 2.9 |
|  | 2019-07 | 16 | 2.5 |
|  | 2019-08 | 21 | 3.3 |
|  | 2019-09 | 16 | 2.5 |
|  | 2019-10 | 25 | 3.9 |
|  | 2019-11 | 21 | 3.3 |
|  | 2019-12 | 17 | 2.6 |
|  | 2020-01 | 24 | 3.7 |
|  | 2020-02 | 22 | 3.4 |
|  | Total | 645 | 100.0 |
| During-COVID | 2020-03 | 15 | 4.2 |
|  | 2020-04 | 9 | 2.5 |
|  | 2020-05 | 13 | 3.6 |
|  | 2020-06 | 4 | 1.1 |
|  | 2020-07 | 13 | 3.6 |
|  | 2020-08 | 9 | 2.5 |
|  | 2020-09 | 15 | 4.2 |
|  | 2020-10 | 21 | 5.8 |
|  | 2020-11 | 25 | 6.9 |
|  | 2020-12 | 21 | 5.8 |
|  | 2021-01 | 18 | 5.0 |
|  | 2021-02 | 16 | 4.4 |
|  | 2021-03 | 22 | 6.1 |
|  | 2021-04 | 20 | 5.5 |
|  | 2021-05 | 12 | 3.3 |
|  | 2021-06 | 19 | 5.3 |
|  | 2021-07 | 23 | 6.4 |
|  | 2021-08 | 25 | 6.9 |
|  | 2021-09 | 17 | 4.7 |
|  | 2021-10 | 17 | 4.7 |
|  | 2021-11 | 10 | 2.8 |
|  | 2021-12 | 17 | 4.7 |
|  | Total | 361 | 100.0 |
| Post-COVID | 2022-01 | 11 | 2.0 |
|  | 2022-02 | 19 | 3.5 |
|  | 2022-03 | 20 | 3.7 |
|  | 2022-04 | 16 | 2.9 |
|  | 2022-05 | 12 | 2.2 |
|  | 2022-06 | 1 | 0.2 |
|  | 2022-07 | 11 | 2.0 |
|  | 2022-08 | 11 | 2.0 |
|  | 2022-09 | 25 | 4.6 |
|  | 2022-10 | 13 | 2.4 |
|  | 2022-11 | 16 | 2.9 |
|  | 2022-12 | 18 | 3.3 |
|  | 2023-01 | 20 | 3.7 |
|  | 2023-02 | 16 | 2.9 |
|  | 2023-03 | 11 | 2.0 |
|  | 2023-04 | 8 | 1.5 |
|  | 2023-05 | 16 | 2.9 |
|  | 2023-06 | 23 | 4.2 |
|  | 2023-07 | 16 | 2.9 |
|  | 2023-08 | 16 | 2.9 |
|  | 2023-09 | 8 | 1.5 |
|  | 2023-10 | 14 | 2.6 |
|  | 2023-11 | 23 | 4.2 |
|  | 2023-12 | 22 | 4.0 |
|  | 2024-01 | 23 | 4.2 |
|  | 2024-02 | 19 | 3.5 |
|  | 2024-03 | 13 | 2.4 |
|  | 2024-04 | 14 | 2.6 |
|  | 2024-05 | 22 | 4.0 |
|  | 2024-06 | 15 | 2.7 |
|  | 2024-07 | 23 | 4.2 |
|  | 2024-08 | 19 | 3.5 |
|  | 2024-09 | 14 | 2.6 |
|  | 2024-10 | 10 | 1.8 |
|  | 2024-11 | 7 | 1.3 |
|  | Total | 545 | 100.0 |

*Counts are monthly admission totals used in the segmented Poisson regression (95 months, January 2017 to November 2024). Percentages are within each COVID period.*

**Supplementary Table 4. Descriptive statistics for length of stay across COVID-19 periods**

| **Measure** | **Statistic** | **Pre-COVID** | **During-COVID** | **Post-COVID** |
| --- | --- | --- | --- | --- |
| LOS (days) | Mean | 26.30 | 24.87 | 29.92 |
|  | Median | 21.00 | 18.00 | 20.00 |
|  | SD | 26.36 | 35.54 | 43.61 |
|  | IQR | 20.00 | 16.00 | 22.50 |
|  | Skewness | 5.66 | 6.80 | 6.54 |
|  | Kurtosis | 58.40 | 56.20 | 56.42 |
| Log LOS | Mean | 1.28 | 1.24 | 1.29 |
|  | Median | 1.32 | 1.26 | 1.30 |
|  | SD | 0.35 | 0.34 | 0.38 |
|  | IQR | 0.43 | 0.39 | 0.48 |
|  | Skewness | -0.21 | 0.25 | 0.13 |
|  | Kurtosis | 0.24 | 1.68 | 0.77 |

*LOS, length of stay; SD, standard deviation; IQR, interquartile range. Log LOS is the natural-log transformation used in the linear mixed-effects model.*

**Supplementary Table 5. Distribution of long hospital stays (>30 days) by COVID-19 period**

| **COVID period** | **LOS category** | **Frequency** | **%** |
| --- | --- | --- | --- |
| Pre-COVID | ≤30 days | 471 | 73.0 |
|  | >30 days | 174 | 27.0 |
|  | Total | 645 | 100.0 |
| During-COVID | ≤30 days | 289 | 80.1 |
|  | >30 days | 72 | 19.9 |
|  | Total | 361 | 100.0 |
| Post-COVID | ≤30 days | 388 | 71.2 |
|  | >30 days | 157 | 28.8 |
|  | Total | 545 | 100.0 |

*Stays longer than 30 days differed across periods (χ²(2) = 9.44; P = .009). Percentages are within each COVID period.*

**Supplementary Figure 1. Distribution of psychiatric diagnostic groups across COVID-19 periods**

*Values are the percentage of admissions in each diagnostic group within each COVID-19 period (overall χ²(8) = 17.93; P = .02; Cramér V = 0.08). Substance use disorders rose from 9.0% pre-COVID to 14.3% post-COVID.*

**Supplementary Figure 2. Length-of-stay categories across COVID-19 periods**

*Percentage of patients with a length of stay of 30 days or less versus more than 30 days, within each COVID-19 period (χ²(2) = 9.44; P = .009).*

**Supplementary Note. Residual serial correlation of the segmented Poisson model.**

Lag-1 autocorrelation of the model residuals was 0.33 and lag-2 was 0.12. Partial autocorrelation at lag 1 was 0.33. The Ljung-Box test over 12 lags was significant (χ² = 31.8; P = .002), indicating low-order autocorrelation. Adding a first-order lag of the monthly count raised the Durbin-Watson statistic from 1.30 to 1.93 but did not fully remove the dependence (Ljung-Box P = .004). Inference was based on Newey-West standard errors, which allow for autocorrelation, with a maximum lag of 3 (level change IRR 0.685; 95% CI 0.51-0.91; P = .01). A generalized estimating equation with a first-order autoregressive working correlation (estimated autocorrelation 0.31) gave a concordant estimate (IRR 0.687; 95% CI 0.54-0.88).

**Supplementary Table 6. Age distribution of psychiatric inpatient admissions across COVID-19 periods.**

| Age group, y | Pre-COVID, No. (%) | During-COVID, No. (%) | Post-COVID, No. (%) |
| --- | --- | --- | --- |
| 18-24 | 124 (19.3) | 64 (17.7) | 101 (18.6) |
| 25-34 | 217 (33.7) | 138 (38.2) | 201 (37.0) |
| 35-44 | 160 (24.8) | 100 (27.7) | 119 (21.9) |
| 45-54 | 96 (14.9) | 43 (11.9) | 67 (12.3) |
| 55-64 | 35 (5.4) | 13 (3.6) | 43 (7.9) |
| 65 or older | 12 (1.9) | 3 (0.8) | 12 (2.2) |

Age distribution did not differ significantly across periods (χ²(10) = 16.77; P = .08).
